# Immediate effects of red (660 nm) and infrared (808 nm) photobiomodulation therapy on fatigue of the orbicularis oris muscle: a randomized clinical study

**DOI:** 10.1590/2317-1782/20212020363

**Published:** 2021-10-22

**Authors:** Mariana Rodrigues Batista, Ludmila Andrade Estrela, Vanessa Mouffron Novaes Alves, Andréa Rodrigues Motta, Renata Maria Moreira Moraes Furlan

**Affiliations:** 1 Programa de pós-graduação em Ciências Fonoaudiológicas, Universidade Federal de Minas Gerais – UFMG - Belo Horizonte (MG), Brasil.; 2 Centro Universitário Metodista Izabela Hendrix – CEUMIH - Belo Horizonte (MG), Brasil.; 3 Departamento de Fonoaudiologia, Universidade Federal de Minas Gerais – UFMG - Belo Horizonte (MG), Brasil.

**Keywords:** Low-Level Light Therapy, Muscle Strength, Lip, Stomatognathic System, Muscle Fatigue

## Abstract

**Purpose:**

To compare the immediate effects of laser photobiomodulation at wavelengths of 660 nm and 808 nm on fatigue of the orbicularis oris.

**Methods:**

This is a randomized study with 60 women aged between 19 and 43 years. The participants were divided into four groups. Group RG received photobiomodulation with a laser wavelength of 660 nm at four points of the orbicularis oris; group IRG received photobiomodulation with a laser wavelength of 808 nm at the same points; the control group did not receive light treatment; and the placebo group underwent the same procedures as RG and IRG but with the equipment switched off. The irradiation was performed with a laser of 100 mW of power, 4 J of energy per point and 133.3 J/cm^2^ of fluency. An electromyography evaluation was performed before and after the irradiation, concomitantly with the exercise of lip protrusion maintained until the sensation of fatigue. Fatigue was evaluated by a median frequency using the electromyographic fatigue index. The amplitude of the signal was evaluated, examining the root mean square, and the values were normalized by the peak. The difference in amplitude between the upper and lower lips was also analyzed. All variables were compared before and after irradiation.

**Results:**

No significant difference was found between the measures taken before and after irradiation.

**Conclusion:**

Photobiomodulation with the parameters investigated in this study had no immediate effect on orbicular oris fatigue.

## INTRODUCTION

The orbicularis oris muscle is actively involved in important functions such as speech, breathing, chewing, swallowing, and facial mimic^(1)^. Some clinical conditions such as mouth breathing, facial palsy, and deleterious oral habits can impair the orbicularis oris muscle, altering the usual position of the lips^(2-4)^ and damaging orofacial functions. In some individuals, lip incompetence influences the position of the incisor teeth, causing tooth movement^(4)^. Therefore, upon detecting lip weakness, it is important to engage in myofunctional therapy with strengthening exercises for the orbicularis oris muscle^(2)^.

Myofunctional therapy helps patients to achieve muscle and functional changes through exercises aimed at correcting muscle condition (myotherapy) and some functions^(2)^. The success of the therapy depends on the therapist’s knowledge of the patient’s individual capacities and on the treatment limitations^(5)^.

Muscle fatigue is among these limitations and refers to the inability of the muscle to maintain an expected level of strength over a period of time^(6,7)^. This is due to a high concentration of some substrates, such as lactic acid, inside muscle cells, which interferes with intracellular pH and hinders conduction of action potentials essential for muscle activation. It is considered a natural defense mechanism of the muscle and is triggered before damage occurs at the cellular and organ levels. Sustaining muscular exercises can lead to pain, discomfort, and interference with motor performance, causing a decrease in therapy functional time^(7)^.

Surface electromyography (EMG) is an objective approach that allows to examine muscle fatigue by assessing the number of activated motor units through signal amplitude, in addition to firing frequency of motor neurons through average frequency (AF) analysis. The process of muscle fatigue involves an increase in the number of activated motor units and a decrease in the firing frequency of motor neurons, resulting in a larger amplitude and lower AF, respectively^(8)^.

The literature indicates that photobiomodulation therapy (PBMT) can delay muscle fatigue during maximum and submaximal contractions^(9-11)^. The benefits of photobiomodulation therapy for muscle tissue also include better muscle performance, greater strength gain, and muscle relaxation^(12-14)^. During the exercise, cells synthetize a great quantity of adenosine triphosphate (ATP) at high speed to supply the energy requirements and prevent fatigue^(15)^. As it increases ATP input, laser is able to increase muscular exercise functional time, thus delaying fatigue.

Some studies assessed the effects of photobiomodulation therapy using infrared wavelength laser on muscle performance and found delay/reduction of muscle fatigue^(13)^. One of these studies^(10)^ observed greater resistance to muscle fatigue in the femoral quadriceps of healthy men irradiated with 808-nm-wavelength laser applied during intervals between series of exercises, as well as after the last series.

Another study^(9)^ found a lower fatigue index in the femoral quadriceps of healthy women who trained for nine consecutive weeks. In this case, a laser with wavelength of 808 nm was applied immediately after each training session^(9)^. Photobiomodulation therapy with a 808-nm-wavelength laser, before exercise, led to a significantly lower dynamometric fatigue index of the plantar flexor muscles in healthy adults compared to both the control and the placebo group^(16)^. When applied immediately before the exercise, the same laser decreased muscle fatigue in the rectus femoris muscle of elderly women^(11)^. However, we found no studies demonstrating the influence of photobiomodulation therapy on lip performance. If the beneficial effect of photobiomodulation on the performance of the orbicularis oris muscle is proven, this therapeutic technique can be used in orofacial myofunctional therapy to optimize the process.

In this context, our objective was to compare the effects of photobiomodulation using laser at the wavelengths of 660 nm (red) and 808 nm (infrared) on the performance of the orbicularis oris muscle in a sustained contraction task. Our hypothesis is that 808-nm-wavelength laser irradiation is more effective for presenting greater depth of penetration into the tissue^(17)^.

## METHODS

This is an experimental, randomized, triple-blind study carried out after approval by the Research Ethics Committee (CAAE 03142818,9,0000,5096) of Centro Universitário Metodista Izabela Hendrix. All procedures performed in this study are in accordance with the ethical standards of the Research Ethics Committee of the institution, as well as the Helsinki Declaration of 1964 and its amendments. All participating individuals signed an Informed Consent Form. This study is registered on ensaiosclinicos.gov.br (RBR-32RP22).

### Sample

The sample was composed of 60 healthy women with an average age of 25 years – minimum age of 19 years, maximum of 43 years, and standard deviation of 5.9. The participants were randomly distributed into four groups of 15 participants each, as follows:

- Group 1 (RG): subjected to low-level laser irradiation at a wavelength of 660-nm (red);
- Group 2 (IRG): subjected to low-level laser irradiation at a wavelength of 808-nm (infrared);
- Control group (CG): not subjected to low-level laser irradiation.
- Placebo group (PG): subjected to the same procedure as the RG and IRG without activating the equipment.

The sample included women aged between 18 and 60 years. The following exclusion criteria were considered: presence of craniofacial anomaly, disease with neuromuscular involvement, regular use of myorelaxant and/or anti-inflammatory drugs, and contraindications for phototherapy — namely, photosensitivity, pregnancy, glaucoma, undiagnosed lesion on or near the area to be irradiated, infection at the application site, cancer history, use of a pacemaker or another electronic implant^(18)^.

### Electromyographic evaluation

Guided by the Frankfurt Plane, each participant was instructed to remain seated on a chair with a 90° angle between the hips, knees and ankles, and with erect posture. The participant’s skin was cleaned using gauze soaked in 70% alcohol at the sites where the electrodes would be placed for the electromyographic evaluation: on the skin above the orbicularis oris muscle, one pair in the upper portion and another in the lower portion. The reference electrode (ground) was positioned on the wrist bone. The electromyographic evaluation was performed concomitantly to the isometric exercise of sustained lip protrusion until the participant felt fatigue, commonly characterized as slight burning, tingling, pain and/or inability to maintain contraction, which the participant was instructed to immediately signal to the researcher.

We recorded the muscle electrical signal using a Miotec® equipment, New Miotool Face model, with two input channels, 16-bit resolution, 3000 V safety isolation, maximum acquisition capacity of 3,000 samples per second, 20 Hz high-pass, and 500 Hz low-pass filters. The Miotec Suite software was used to collect and analyze the data on a laptop computer that was not connected to the mains. For data collection, we used circular, double-sided surface sensors made of Ag/AgCl material with Miotec® fixed conductive gel. The electrodes had a diameter of 10 mm and the distance between each other was fixed at 20 mm. Equipment gain was automatic. Input impedance was 10 GΩ and the common mode rejection ratio was >100 dB.

**Figure 1:**
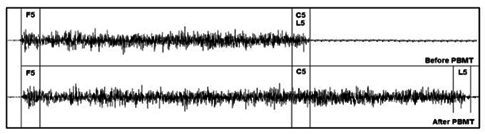
Schematic drawing of the electromyographic signal and sections analyzed. F5 = first section of 5 s; L5 = last section of 5 s; C5 = last section of 5 s shared by both signals

To analyze the signal, we discarded the first seconds before the beginning of muscle activity, considered as the moment when the electric signal amplitude exceeded the average increased by two standard deviations of the signal obtained at rest^(8)^. From the beginning of the activity, we disregarded the 0.5 initial seconds to homogenize the analyzed sections. The signal was divided into sections of 5 s, which were analyzed in the AF domain (through fast Fourier transform): first 5 seconds (F5), last 5 seconds (L5), and last section of 5 seconds with duration common to both signals (C5). As the electric signal of the lips was obtained before and after the PBMT, with different durations, this last parameter was required to ensure that the comparison of signals before and after the laser application was performed after the same muscle contraction time, as illustrated in Figure 1.

We also analyzed the values of electric signal amplitude in RMS and normalized by the signal peak by comparing with the pre- and post-laser stages. The difference in signal amplitude between the upper and lower lips was obtained to examine a possible alteration in muscle balance. Finally, we calculated the electromyographic fatigue index (EFI) by dividing the values of final FM by initial FM.

We compared the following variables regarding the moments before and after PBMT: (a) EFI calculated using the formula EFI=L5/F5^(11,16)^; (b) EFI calculated using the formula EFI=C5/F5; (c) values of RMS amplitude in the signal, in µV; (d) amplitude normalized by the peak, and (e) difference in electric signal amplitude between the upper and lower lips.

### Application of low-level laser

After the initial electromyographic evaluation, we performed a low-level laser irradiation using a MMOptics® equipment (São Carlos – SP, Brazil). Chart 1 lists the irradiation parameters applied. Before starting the experiments, the laser equipment was calibrated by the manufacturer.

**Chart 1:** Laser parameters

Irradiation parameters	Values
Wavelength	660 nm (red) or 808 nm (infrared)
Operation mode	continuous
Optical output	100 mW
Exit spot diameter	1.95 mm
Exit spot area	0.03 cm^2^
Power density	3.3 W/cm^2^
Energy per point	4 J
Energy density (fluence) per point	133.3 J/cm^2^
Application time per point	40 s
Number of points	4
Total energy	16 J
Application mode	Stationary mode of contact

**Figure 2:**
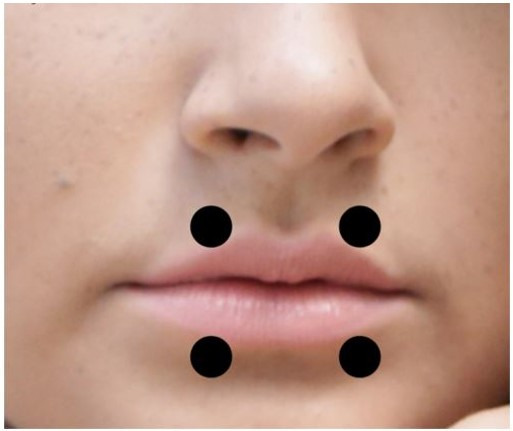
Laser application points

We applied the laser on four points of the orbicularis oris muscle: two points on the upper lip and two points on the lower lip (Figure 2) at a dose of 4 J per point, resulting in a total dose of 8 J for the upper and 8 J for the lower lip. For the PG, the equipment was positioned at the same points as for the experimental groups, turned on to emit sound, but not activated. Although the CG did not receive PBMT, we respected the same time interval between irradiation and electromyographic evaluation used for the other groups.

In all groups, irradiation was performed by placing the tip of the equipment against the skin of the participant. The device was sanitized with 70% alcohol before each application and the tip was covered with a transparent plastic film, which was changed with each new participant. During the irradiation procedure, both researchers and participants used goggles supplied by the equipment manufacturer.

The researcher who applied the laser was not the same who carried out the electromyographic evaluation; the latter was blind to the group to which the individual belonged. The participants were also not aware of which group they were part of. The electric signals were analyzed by a third researcher, who was also blind to the group each participant belonged to and was not informed if the signal analyzed referred to a collection before or after irradiation.

Following irradiation, the participants were given a five-minute rest period, after which the electromyographic evaluation procedures were repeated.

### Data analysis

We used the Kolmogorov-Smirnov statistical test to assess the distribution of the variables in the study. Since the variables did not present normal distribution, the analyses were performed through non-parametric tests. The Kruskal-Wallis test was applied to compare the dosages among the groups, while the Wilcoxon test compared the variables before and after the laser intervention. All tests were performed at a significance level of 5%.

## RESULTS

The results indicated no statistically relevant difference by comparing the groups regarding age (Table 1).

**Table 1:** Comparison of the participants’ age among the groups

Group		Age	Value of p^*^
CG (n=15)	Average	25.4	0.254
	SD	7.1	
	Median	22.0	
	Minimum	19.0	
	Maximum	42.0	
RG (n=15)	Average	23.4	
	SD	4.4	
	Median	22.0	
	Minimum	19.0	
	Maximum	35.0	
IRG (n=15)	Average	26.8	
	SD	7.3	
	Median	25.0	
	Minimum	19.0	
	Maximum	43.0	
PG (n=15)	Average	24.5	
	SD	3.9	
	Median	23.0	
	Minimum	20.0	
	Maximum	34.0	

* Kruskal-Wallis Test

**Caption:** CG = control group; RG = group irradiated with red laser; IRG = group irradiated with infrared laser; PG = placebo group; SD = standard deviation; n = number of subjects.

Comparative analysis of the electromyographic fatigue indices before and after PBMT indicated no statistically significant difference for the upper and lower lips in any of the groups (Table 2).

**Table 2:** Comparison of electromyographic fatigue index before and after photobiomodulation

Electrode site			EFI (C5/F5)		Before x After Value of p^*^	EFI (L5/F5)		Before x After Value of p*
			Before PBMT	After PBMT		Before PBMT	After PBMT	
Upper lip	CG (n=15)	Average	0.88	0.89	1.000	0.88	0.87	0.865
		SD	0.10	0.04		0.09	0.05	
		Median	0.87	0.91		0.86	0.87	
		Minimum	0.69	0.82		0.75	0.77	
		Maximum	1.08	0.95		1.08	0.98	
	RG (n=15)	Average	0.87	0.83	0.363	0.85	0.82	0.532
		SD	0.17	0.09		0.16	0.10	
		Median	0.89	0.83		0.86	0.85	
		Minimum	0.35	0.66		0.35	0.66	
		Maximum	1.14	0.98		1.05	0.99	
	IRG (n=15)	Average	0.88	0.86	0.140	0.87	0.87	0.609
		SD	0.05	0.05		0.06	0.06	
		Median	0.89	0.85		0.86	0.85	
		Minimum	0.75	0.78		0.75	0.78	
		Maximum	0.95	0.99		0.98	0.97	
	PG (n=15)	Average	0.89	0.90	0.460	0.90	0.87	0.334
		SD	0.08	0.09		0.08	0.08	
		Median	0.87	0.88		0.91	0.88	
		Minimum	0.79	0.79		0.79	0.75	
		Maximum	1.10	1.07		1.10	1.07	
Lower lip	CG (n=15)	Average	0.91	0.98	0.363	0.90	0.90	0.609
		SD	0.16	0.31		0.16	0.18	
		Median	0.89	0.88		0.88	0.86	
		Minimum	0.63	0.72		0.62	0.72	
		Maximum	1.28	1.88		1.28	1.52	
	RG (n=15)	Average	0.85	0.93	0.691	0.85	0.92	0.955
		SD	0.13	0.25		0.12	0.26	
		Median	0.86	0.88		0.84	0.88	
		Minimum	0.64	0.73		0.65	0.71	
		Maximum	1.09	1.80		1.09	1.80	
	IRG (n=15)	Average	0.94	0.84	0.053	0.94	0.84	0.064
		SD	0.25	0.06		0.26	0.07	
		Median	0.91	0.83		0.90	0.82	
		Minimum	0.53	0.76		0.71	0.76	
		Maximum	1.74	1.03		1.82	1.02	
	PG (n=15)	Average	0.92	0.86	0.570	0.92	0.83	0.281
		SD	0.21	0.09		0.21	0.10	
		Median	0.88	0.86		0.87	0.86	
		Minimum	0.74	0.74		0.74	0.63	
		Maximum	1.59	1.03		1.59	0.99	

* Wilcoxon Test

**Caption:** CG = control group; RD = group irradiated with red laser; IRF = group irradiated with infrared laser; PG = placebo group; EFI = electromyographic fatigue index; PBMT = photobiomodulation therapy; SD = standard deviation; n = number of subjects.

The average values of RMS and normalized by the peak amplitudes did not indicate any statistically significant difference compared before and after the PBMT (Table 3).

**Table 3:** Comparison of the average values of RMS and amplitude normalized by the peak before and after photobiomodulation

Site of electrode			Average (RMS)			Values normalized by the peak (%)		
			Before PBMT	After PBMT	Before x After p value^*^	Before PBMT	After PBMT	Before x After p value*
Upper lip	CG (n=15)	Average	110.10	107.64	0.532	68.94	68.14	0.910
		DP	36.57	52.69		9.53	10.19	
		Median	101.94	109.84		69.71	70.64	
		Minimum	53.92	27.93		51.01	47.96	
		Maximum	176.21	199.43		82.74	84.24	
	RG (n=15)	Average	145.61	138.21	0.733	67.94	65.25	0.470
		DP	87.17	67.60		6.56	7.90	
		Median	134.85	116.60		67.25	66.20	
		Minimum	25.46	37.67		55.44	44.74	
		Maximum	309.66	263.43		77.02	75.54	
	IRG (n=15)	Average	119.80	120.23	0.955	66.05	66.63	0.865
		DP	49.98	47.55		10.11	5.25	
		Median	111.86	133.43		67.90	64.96	
		Minimum	43.21	41.59		45.23	60.07	
		Maximum	195.68	209.90		82.26	75.40	
	PG (n=15)	Average	117.93	115.53	0.496	67.12	64.97	0.394
		DP	79.11	67.10		7.38	9.32	
		Median	118.17	122.36		68.19	67.08	
		Minimum	26.62	21.78		53.07	42.12	
		Maximum	308.36	221.91		77.55	75.51	
Lower lip	CG (n=15)	Average	212.51	229.46	0.281	66.58	68.11	0.865
		DP	76.01	66.10		12.02	8.38	
		Median	205.19	235.74		70.32	68.42	
		Minimum	105.24	111.49		31.16	51.11	
		Maximum	364.98	329.42		76.09	80.26	
	RG (n=15)	Average	279.60	269.90	0.701	67.51	66.65	0.638
		DP	131.33	109.33		12.57	8.46	
		Median	309.45	238.77		70.98	66.46	
		Minimum	93.43	117.86		32.64	44.65	
		Maximum	466.10	463.74		78.30	81.33	
	IRG (n=15)	Average	196.12	226.80	0.233	71.40	63.30	0.820
		DP	79.87	91.51		39.17	17.01	
		Median	213.34	214.28		64.18	66.97	
		Minimum	56.58	99.28		18.24	13.30	
		Maximum	321.14	473.99		202.54	79.78	
	PG (n=15)	Average	217.92	212.57	0.650	63.96	65.94	0.865
		DP	132.07	105.65		14.00	7.52	
		Median	158.68	218.37		69.50	66.79	
		Minimum	35.74	64.26		25.26	53.64	
		Maximum	452.33	386.26		78.98	81.62	

* Wilcoxon Test

**Caption:** RMS = Root Mean Square; CG = control group; RG = group irradiated with red laser; IRG = group irradiated with infrared laser; PG = placebo group; PBMT = photobiomodulation therapy; SD = standard deviation, n = number of subjects

Additionally, no statistically relevant differences were found in the comparison analysis between the upper and lower lips before and after the PBMT (Table 4).

**Table 4:** Difference between the amplitude of lower and upper lips before and after photobiomodulation

Site of electrode		Difference between upper and lower lips (RMS)		p value^*^
		Before PBMT	After PBMT	
CG (n=15)	Average	113.71	124.45	0.394
	DP	69.56	70.33	
	Median	99.58	123.57	
	Minimum	3.91	3.92	
	Maximum	227.76	210.03	
RG (n=15)	Average	148.39	131.68	0.173
	DP	81.71	70.81	
	Median	156.60	121.37	
	Minimum	10.86	16.70	
	Maximum	313.73	254.37	
IRG (n=15)	Average	82.00	111.68	0.307
	DP	56.00	97.40	
	Median	94.19	88.46	
	Minimum	0.45	17.88	
	Maximum	174.05	399.86	
PG (n=15)	Average	127.05	114.93	0.532
	DP	85.15	61.43	
	Median	114.49	92.06	
	Minimum	5.56	24.49	
	Maximum	302.20	202.00	

* Wilcoxon Test

**Caption:** RMS = Root Mean Square; CG = control group; RG = group irradiated with red laser; IRG = group irradiated with infrared laser; PG = placebo group; PBMT = photobiomodulation therapy; SD = standard deviation; n = number of subjects.

## DISCUSSION

Our study did not find any immediate effects of PBMT on electromyographic fatigue of the orbicularis oris muscle. So far, studies assessing the effects of photobiomodulation on this musculature were not found.

The variable gender of the participants could have influenced the remaining variables, as men usually have greater lip strength than women^(19,20)^. Therefore, we decided to include only female participants in the sample. The statistical analysis demonstrated that the tested groups were homogeneous regarding age, which is relevant since lip strength is also influenced by age^(21)^. Our choice for the dose of 4 J was based on previous studies addressing the effects of PBMT on the performance of different muscles^(10,22)^.

de Almeida et al.^(23)^ studied the effects of red (660 nm) and infrared (830 nm) lasers on the performance of the biceps brachii muscle and found that both lengths had positive effects. However, only infrared laser influenced fatigue. According to the authors, such difference results from an increased penetration range of the infrared laser, which is able to reach deeper fibers, while red wavelength laser acts more superficially^(23)^. Since light penetration increases as the wavelength laser increases^(17)^, infrared laser is more widely used in studies addressing muscle performance analysis^(13)^. However, as the orbicularis oris is a superficially located fine^(24)^ muscle^(1)^, we considered important to observe not only the effects of the infrared but also of the red laser on this muscle.

PBMT at both wavelengths was not able to influence the parameters related to electromyographic signal amplitude (RMS and normalized amplitudes), thus corroborating some previous studies^(25,26)^. da Silva Alves et al.^(25)^ did not observe any effect of infrared laser (850 nm) on the electromyographic signal amplitude of the quadriceps and gastrocnemius muscles in young men, while dos Santos Maciel et al.^(26)^ found no influence of 780-nm laser irradiation before strength and resistance exercises on the RMS of anterior tibial muscles, measured during the exercises. However, Muñoz et al.^(27)^ observed an increase in electromyographic signal amplitude after infrared laser (780 nm) irradiation on the masseter muscle of healthy men^(27)^ at a dose of 0.8 J per point at 8 irradiated points. The differences in the dosimetry parameters and muscles assessed did not enable comparison of these studies’ results.

PBMT has demonstrated positive results when interacting with biological tissues, promoting an increase in the production of cellular energy, especially due to the absorption of light energy by mitochondria, thus stimulating the respiratory chain^(11)^. Such interaction alters the redox potential of the cytoplasm and accelerates the flow in the mitochondrial electron transport chain, increasing ATP synthesis^(11)^, which explains the improved muscle performance after PBMT found in some studies^(23,28,29)^. A possible explanation for our results concerns the short time period between laser application and data collection, which is insufficient for the light to interact with the tissue. The existence of a dose-response curve reported in the irradiation parameters could be another explanation. The laser energy applied to the mouth orbicular may have been insufficient to improve muscle performance.

In this study, regardless of the wavelength, PBMT did not have any effect on electromyographic fatigue, corroborating some studies^(29,30)^ and contradicting others^(9,11,16)^. After subjecting the biceps brachii muscle of young women to a 808-nm- wavelength laser irradiation before the fatigue protocol, Higashi et al.^(30)^ did not observe any effect of the laser on electromyographic fatigue. Similarly, Toma et al.^(29)^ demonstrated that applying PBMT using 808-nm laser after strengthening exercises for the quadriceps of elderly individuals did not alter the electromyographic fatigue index. However, another study^(11)^ found that PBMT (808 nm) carried out immediately before exercises for the rectus femoris muscle significantly reduced the electromyographic fatigue index. After subjecting the femoral quadriceps muscle to a 808-nm laser irradiation between and after exercises for two days, de Brito Vieira et al.^(9)^ verified a decrease in electromyographic fatigue for the vastus medialis and rectus femoris in relation to the placebo group, suggesting an influence of post-irradiation time on reduced muscle fatigue.

de Souza et al.^(16)^ applied an isokinetic dynamometry and found that the PBMT (808 nm) before the exercise reduced fatigue in the ankle plantar flexors of healthy individuals, but did not alter the average frequency of the surface electromyography. The authors suggest that the energy source of the primary action can be related to energy production inside the muscle fibers rather than to the influence of neuromuscular recruitment. The evolution of fatigue encompasses several factors, such as alteration in motor unit recruitment, decrease in electric potential of the membrane, and increase in levels of reactive oxygen species and reactive nitrogen species. Although average frequency is regarded as a satisfactory parameter for the electromyographic analysis of neuromuscular fatigue^(16)^, it reflects the firing rate of neuromuscular action potential. The lack of difference in the average frequency found in this study can be related to the absence of PBMT influence on the neuromuscular recruitment pattern.

Our study also assessed the difference in signal amplitude between the lower and upper lips for considering that it influences the balance between the muscles after PBMT; however, such effect could not be proven. No studies were found to address this variable for comparison.

The limitations of this study include short rest time between exercises, as well as between irradiation and exercise, and lack of control of anatomical characteristics, such as lip thickness, which is likely to influence the results. For this reason, our comparisons were exclusively intragroup. We suggest that further studies use different doses and include individuals with orofacial myofunctional alterations, such as mouth breathers.

## CONCLUSION

We found no differences in the surface electromyography between the measures of average frequency and signal amplitude performed before and after mouth orbicular irradiation with low-level laser using the wavelengths of 660 nm and 830 nm. Therefore, photobiomodulation based on the parameters assessed in this study did not result in immediate effects on the fatigue of mouth orbicular.
