# Supplementary material for: Immediate effects of red (660 nm) and infrared (808 nm) photobiomodulation therapy on fatigue of the orbicularis oris muscle: a randomized clinical study
Source: Codas. 2021 Oct 22;34(2):e20200363. doi: 10.1590/2317-1782/20212020363 (PMC9851187; doi:10.1590/2317-1782/20212020363)
Supplement: Translation

# Translation: Efeitos imediatos da fotobiomodulação com laser de baixa intensidade e comprimentos de onda vermelho (660 nm) e infravermelho (808 nm) na fadiga eletromiográfica do músculo orbicular da boca: estudo clínico randomizado

**Authors:** Mariana Rodrigues Batista, Ludmila Andrade Estrela, Vanessa Mouffron Novaes Alves, Andréa Rodrigues Motta, Renata Maria Moreira Moraes Furlan

**Affiliations:** 1 Programa de pós-graduação em Ciências Fonoaudiológicas, Universidade Federal de Minas Gerais – UFMG - Belo Horizonte (MG), Brasil.; 2 Centro Universitário Metodista Izabela Hendrix – CEUMIH - Belo Horizonte (MG), Brasil.; 3 Departamento de Fonoaudiologia, Universidade Federal de Minas Gerais – UFMG - Belo Horizonte (MG), Brasil.

**Keywords:** Terapia com Luz e Baixa Intensidade, Força Muscular, Lábio, Sistema Estomatognático, Fadiga Muscular

## Abstract

**Objetivo:**

comparar os efeitos imediatos da fotobiomodulação com laser nos comprimentos de onda 660 nm e 808 nm na fadiga do músculo orbicular da boca.

**Métodos:**

trata-se de um estudo experimental randomizado, com 60 mulheres, com idade entre 19 e 43 anos. As participantes foram divididas em quatro grupos. O grupo GV recebeu irradiação com laser de comprimento de onda de 660 nm em quatro pontos do orbicular da boca; o grupo GIV recebeu irradiação com laser de comprimento de onda de 808 nm nos mesmos pontos; o grupo controle não recebeu irradiação e o grupo placebo passou pelos mesmos procedimentos dos grupos GV e GIV, porém o equipamento não foi acionado. A irradiação foi realizada com laser de 100 mW de potência, 4 J de energia por ponto e 133,3 J/cm2 de fluência. Avaliação eletromiográfica foi realizada antes e após a irradiação, concomitantemente ao exercício de protrusão labial sustentada até a sensação de fadiga. O índice de fadiga eletromiográfica foi calculado a partir da frequência mediana. A amplitude do sinal foi avaliada, examinando o RMS, e os valores normalizados pelo pico. A diferença na amplitude entre lábios superiores e inferiores também foi analisada. Todas as variáveis foram comparadas antes e após a irradiação.

**Resultados:**

não foram encontradas diferenças significativas nas medidas antes e após a irradiação.

**Conclusão:**

a fotobiomodulação, com os parâmetros investigados neste estudo, não resultou em efeitos imediatos sobre a fadiga do músculo orbicular da boca.

## INTRODUÇÃO

O músculo orbicular da boca está envolvido ativamente em importantes funções como fala, respiração, mastigação, deglutição e mímica facial^(1)^. Algumas condições clínicas, tais como respiração oral, paralisia facial e hábitos orais deletérios, podem enfraquecer o músculo orbicular da boca, causando alteração na postura habitual dos lábios^(2-4)^ e prejuízo para as funções orofaciais. Em alguns indivíduos, a incompetência labial influencia a posição dos dentes incisivos, gerando movimentação dentária^(4)^. Sendo assim, quando a fraqueza labial é detectada, exercícios para fortalecimento do músculo orbicular da boca por meio da terapia miofuncional fazem-se necessários^(2)^.

A terapia miofuncional auxilia o paciente a alcançar mudanças musculares e funcionais, por meio de exercícios para modificação da condição muscular (mioterapia) e do trabalho direcionado para as funções que precisam ser corrigidas^(2)^. O sucesso da terapia depende do conhecimento do terapeuta em relação às capacidades individuais e limitações do tratamento^(5)^.

Uma dessas limitações, a fadiga muscular, refere-se à inabilidade do músculo para manter um nível de força esperado durante um determinado período de tempo^(6,7)^. Isso ocorre devido à alta concentração de alguns substratos no interior das células musculares, tais como ácido lático, que interfere no pH intracelular, dificultando a condução dos potenciais de ação essenciais para a ativação muscular. É considerada um mecanismo de defesa natural do músculo, acionado antes que ocorram danos a níveis celulares e orgânicos. A sustentação de exercícios musculares pode acarretar dores, desconforto e interferência no desempenho motor, causando uma diminuição no tempo funcional da terapia^(7)^.

Um método objetivo que possibilita a investigação da fadiga muscular é a eletromiografia de superfície (EMG). A EMG permite avaliar o número de unidades motoras ativadas, por meio da amplitude do sinal, e a frequência de disparo dos neurônios motores, pela análise da frequência mediana (FM). No processo de fadiga muscular, ocorre o aumento do número de unidades motoras ativadas e diminuição da frequência de disparo dos neurônios motores, resultando em uma maior amplitude e menor FM, respectivamente^(8)^.

A literatura indica que a terapia por fotobiomodulação (TFBM) pode retardar a fadiga muscular durante contração máxima e submáxima^(9-11)^. Os benefícios da terapia por fotobiomodulação para o tecido muscular incluem, ainda, melhor desempenho muscular, maior ganho de força e relaxamento da musculatura^(12-14)^. Durante o exercício, as células sintetizam grande quantidade de adenosina trifosfato (ATP), em velocidade acelerada, para suprir as necessidades energéticas e evitar a fadiga^(15)^. O laser aumenta o aporte de ATP, por isso é capaz de aumentar o tempo funcional do exercício muscular, retardando assim a fadiga.

Alguns estudos avaliaram os efeitos da terapia por fotobiomodulação, usando laser com comprimento de onda infravermelho, no desempenho muscular e verificaram retardo/diminuição da fadiga muscular^(13)^. Um estudo^(10)^ encontrou aumento da resistência à fadiga muscular do quadríceps femoral de homens saudáveis quando irradiados com laser com comprimento de onda de 808 nm, aplicado durante intervalos entre séries de exercícios e após a última série. Outro estudo^(9)^ verificou diminuição no índice de fadiga do quadríceps femoral de mulheres saudáveis que treinaram por nove semanas consecutivas. Nesse caso, um laser de comprimento de onda de 808 nm foi aplicado imediatamente após cada sessão de treinamento^(9)^. A terapia por fotobiomodulação, com laser com comprimento de onda de 808 nm, antes do exercício, provocou diminuição significativa do índice de fadiga dinamométrica dos músculos flexores plantares em adultos saudáveis quando comparados aos grupos controle e placebo^(16)^. O mesmo laser, aplicado imediatamente antes do exercício, reduziu a fadiga muscular do músculo reto femoral em mulheres idosas^(11)^. No entanto, não foram encontrados estudos que comprovassem a influência da terapia por fotobiomodulação no desempenho dos lábios. Se comprovado o efeito benéfico da fotobiomodulação no desempenho do músculo orbicular da boca, esta modalidade terapêutica poderá ser utilizada na terapia miofuncional orofacial, para otimizar o processo terapêutico.

Diante disso, o objetivo do presente estudo foi comparar os efeitos da fotobiomodulação com laser nos comprimentos de onda de 660 nm (vermelho) e 808 nm (infravermelho) no desempenho do músculo orbicular da boca em tarefa de contração sustentada. A hipótese levantada foi que a irradiação com laser com comprimento de onda de 808 nm seria mais efetiva, visto que apresenta maior profundidade de penetração no tecido^(17)^.

## MÉTODO

Trata-se de um estudo experimental, randomizado, triplo-cego, realizado após a aprovação do Comitê de Ética em Pesquisa (sob o número CAAE 03142818.9.0000.5096) do Centro Universitário Metodista Izabela Hendrix. Todos os procedimentos realizados nesta pesquisa estão de acordo com padrões éticos do Comitê de Ética em Pesquisa da instituição e com a Declaração de Helsinki de 1964 e suas emendas. Todos os indivíduos envolvidos assinaram o Termo de Consentimento Livre e Esclarecido. O estudo foi registrado em ensaiosclinicos.gov.br (RBR-32RP22).

### Amostra

A amostra foi composta por 60 mulheres saudáveis, com média de idade de 25 anos, idade mínima de 19 anos, máxima de 43 anos e desvio-padrão de 5,9. As participantes foram alocadas de forma randomizada em quatro grupos, com 15 participantes cada, da seguinte forma:

- Grupo 1 (GV): foi submetido à irradiação com *laser* de baixa intensidade e comprimento de onda de 660 nm (vermelho);
- Grupo 2 (GIV): foi submetido à irradiação com *laser* de baixa intensidade e comprimento de onda de 808 nm; (infravermelho);
- Grupo Controle (GC): não foi submetido à irradiação com *laser* de baixa intensidade;
- Grupo Placebo (GP): foi submetido ao mesmo procedimento do GV e GIV, porém, o equipamento não foi acionado.

Foram incluídas na amostra, mulheres com idade entre 18 e 60 anos. Constituíram os critérios de exclusão: possuir anomalia craniofacial, doença com comprometimento neuromuscular, uso regular de medicamentos miorrelaxantes e/ou anti-inflamatórios, apresentar contraindicações para a fototerapia, sendo elas fotossensibilidade, gravidez, glaucoma, lesão sem diagnóstico sobre a área a ser irradiada ou próxima a ela, infecção no local da aplicação, histórico de câncer, uso de marcapasso ou outro implante eletrônico^(18)^.

### Avaliação eletromiográfica

Cada participante foi instruída a permanecer sentada em uma cadeira, com ângulo de 90° entre quadril, joelhos e tornozelos, postura ereta, guiada pelo Plano de Frankfurt. Foi realizada a limpeza da pele da participante, utilizando gaze embebida em álcool 70% nos locais em que seriam colocados os eletrodos para a avaliação eletromiográfica: sobre a pele acima do músculo orbicular da boca, sendo um par na porção superior e outro par na porção inferior. O eletrodo referência (terra) foi posicionado no osso do punho. A avaliação eletromiográfica foi realizada concomitantemente ao exercício isométrico de protrusão labial sustentada até a participante ter a sensação de fadiga, comumente caracterizada como ligeiro ardor, formigamento, dor e/ou incapacidade de manter a contração, sendo que a participante foi orientada a sinalizar imediatamente este momento ao pesquisador.

O registro do sinal elétrico muscular foi realizado utilizando o equipamento da marca Miotec®, modelo New Miotool Face, com dois canais de entrada, resolução de 16 bits, isolamento de segurança de 3000 V, capacidade de aquisição máxima de 3000 amostras por segundo, filtros passa alta de 20 Hz e passa baixa de 500 Hz. O software Miotec Suite foi utilizado para coleta e análise dos dados em um notebook que não estava conectado à rede elétrica. Para captação dos dados foram utilizados sensores superficiais circulares, do tipo double, de material Ag/AgCl, com gel condutor fixo, sendo a marca Miotec®. Os eletrodos apresentavam diâmetro de 10 mm e a distância entre os eletrodos era fixa de 20 mm. O ganho do equipamento era automático. A impedância de entrada era de 10 GΩ e a taxa de rejeição de modo comum >100 dB.

Para análise do sinal, os primeiros segundos anteriores ao início da atividade muscular foram excluídos, considerando-se como início da atividade muscular o momento em que a amplitude do sinal elétrico ultrapassou a média acrescida de dois desvios padrão do sinal obtido em repouso^(8)^. A partir do início da atividade, foram desconsiderados os 0,5 s iniciais a fim de homogeneizar os trechos analisados. O sinal foi dividido em trechos de 5 s, sendo analisados, no domínio da FM (por transformada rápida de Fourier): os primeiros 5 segundos (F5), os últimos 5 segundos (L5) e o último trecho de 5 segundos com duração comum aos dois sinais (C5). Uma vez que, o sinal elétrico dos lábios foi obtido antes e após a TFBM, tendo diferentes durações, este último parâmetro foi necessário para garantir que a comparação de sinais antes e após a aplicação do *laser* fosse realizada após o mesmo tempo de contração muscular, como ilustrado na Figura 1.

**Figura 1:** Desenho esquemático do sinal eletromiográfico e dos trechos analisados. F5 = primeiro trecho de 5 s; L5 = último trecho de 5 s; C5 = último trecho de 5 s comum aos dois sinais

Também foram analisados os valores de amplitude do sinal elétrico em RMS e normalizados pelo pico do sinal, comparando-se os momentos pré e pós *laser*. A diferença de amplitude do sinal entre os lábios superior e inferior foi obtida para a investigação de uma possível mudança no equilíbrio muscular. Por fim, calculou-se o índice de fadiga eletromiográfica (IFE), por meio da divisão entre os valores da FM final pela inicial.

As seguintes variáveis foram comparadas entre os momentos anterior e posterior à TFBM: (a) o IFE calculado usando a fórmula IFE=L5/F5^(11,16)^; (b) o IFE calculado usando a fórmula IFE=C5/F5; (c) os valores de amplitude RMS do sinal, em µV; (d) a amplitude normalizada pelo pico; e (e) a diferença da amplitude do sinal elétrico entre lábios superior e inferior.

### Aplicação do *laser* de baixa intensidade

Após a avaliação eletromiográfica inicial, foi realizada a irradiação com *laser* de baixa intensidade, utilizando equipamento da marca MMOptics® (São Carlos – SP, Brasil). Os parâmetros de irradiação utilizados estão descritos no Quadro 1. Antes de iniciar os experimentos, o equipamento de *lase*r foi calibrado pelo fabricante.

**Quadro 1:** Parâmetros do *laser*

Parâmetros de irradiação	Valores
Comprimento de onda	660 nm (vermelho) ou 808 nm (infravermelho)
Modo de operação	contínuo
Potência	100 mW
Diâmetro do spot de saída	1,95 mm
Área do spot de saída	0,03 cm^2^
Densidade de potência	3,3 W/cm^2^
Energia por ponto	4 J
Densidade de energia (fluência) por ponto	133,3 J/cm^2^
Tempo de aplicação por ponto	40 s
Número de pontos	4
Energia total	16 J
Modo de aplicação	Modo estacionário com contato

O *laser* foi aplicado em quatro pontos no músculo orbicular da boca: dois pontos no lábio superior e dois pontos no lábio inferior (Figura 2). A dose foi de 4 J por ponto, resultando em dose total de 8 J no lábio superior e 8 J no inferior. No GP, o equipamento foi posicionado nos mesmos pontos que os grupos experimentais, sendo ligado para emitir som, mas não foi acionado. O GC não recebeu TFBM, porém foi respeitado o mesmo intervalo de tempo entre a irradiação e a avaliação eletromiográfica despendido nos demais grupos.

**Figura 2:** Pontos de aplicação do *laser*

Em todos os grupos, a irradiação foi feita encostando a ponteira do equipamento sobre a pele do participante. O aparelho foi higienizado com álcool 70%, antes de cada aplicação e a ponta encapada com um filme plástico transparente, sendo este trocado a cada participante. Durante o procedimento de irradiação, tanto os pesquisadores quanto as participantes utilizaram óculos de proteção fornecidos pelo fabricante do equipamento.

O pesquisador que aplicou o *laser* não foi o mesmo que realizou a avaliação eletromiográfica. Este último não soube de qual grupo o indivíduo participava. O participante não sabia de qual grupo ele fazia parte e a análise dos sinais elétricos foi realizada por um terceiro pesquisador, que também não sabia a qual grupo cada participante pertencia e nem se o sinal analisado referia-se à coleta anterior ou posterior à irradiação.

Após a irradiação, aos participantes foi concedido um período de descanso de cinco minutos, após o qual, os procedimentos de avaliação eletromiográfica foram repetidos.

### Análise dos dados

Aplicou-se o teste estatístico Kolmogorov-Smirnov com a finalidade de avaliar a distribuição das variáveis do estudo. As variáveis não apresentaram distribuição normal e por isso as análises foram feitas por meio de testes não paramétricos. Para comparação das idades entre os grupos utilizou-se o teste Kruskal-Wallis. Para comparação das variáveis antes e após a intervenção do *laser* utilizou-se o teste Wilcoxon. Todos os testes foram realizados com nível de significância de 5%.

## RESULTADOS

Os resultados indicaram ausência de diferença com relevância estatística ao comparar os grupos quanto à idade (Tabela 1).

**Tabela 1:** Comparação da idade dos participantes entre os grupos

Grupo		Idade	Valor de p*
GC (n=15)	Média	25,4	0,254
	DP	7,1	
	Mediana	22,0	
	Mínimo	19,0	
	Máximo	42,0	
GV (n=15)	Média	23,4	
	DP	4,4	
	Mediana	22,0	
	Mínimo	19,0	
	Máximo	35,0	
GIV (n=15)	Média	26,8	
	DP	7,3	
	Mediana	25,0	
	Mínimo	19,0	
	Máximo	43,0	
GP (n=15)	Média	24,5	
	DP	3,9	
	Mediana	23,0	
	Mínimo	20,0	
	Máximo	34,0	

* Teste Kruskal-Wallis

**Legenda:** GC = grupo controle; GV = grupo irradiado com *laser* vermelho; GIV = grupo irradiado com *laser* infravermelho; GP = grupo placebo; DP = desvio padrão; n = número de indivíduos.

A análise comparativa dos índices de fadiga eletromiográfica antes e depois da TFBM não indicou diferença estatisticamente significativa nos lábios superior e inferior em nenhum dos grupos (Tabela 2).

**Tabela 2:** Comparação do índice de fadiga eletromiográfica antes e após a fotobiomodulação

Local do eletrodo			IFE (C5/F5)		Antes x Após Valor de p^*^	IFE (L5/F5)		Antes x Após Valor de p*
			Antes da TFBM	Após a TFBM		Antes da TFBM	Após a TFBM	
Lábio superior	GC (n=15)	Média	0,88	0,89	1,000	0,88	0,87	0,865
		DP	0,10	0,04		0,09	0,05	
		Mediana	0,87	0,91		0,86	0,87	
		Mínimo	0,69	0,82		0,75	0,77	
		Máximo	1,08	0,95		1,08	0,98	
	GV (n=15)	Média	0,87	0,83	0,363	0,85	0,82	0,532
		DP	0,17	0,09		0,16	0,10	
		Mediana	0,89	0,83		0,86	0,85	
		Mínimo	0,35	0,66		0,35	0,66	
		Máximo	1,14	0,98		1,05	0,99	
	GIV (n=15)	Média	0,88	0,86	0,140	0,87	0,87	0,609
		DP	0,05	0,05		0,06	0,06	
		Mediana	0,89	0,85		0,86	0,85	
		Mínimo	0,75	0,78		0,75	0,78	
		Máximo	0,95	0,99		0,98	0,97	
	GP (n=15)	Média	0,89	0,90	0,460	0,90	0,87	0,334
		DP	0,08	0,09		0,08	0,08	
		Mediana	0,87	0,88		0,91	0,88	
		Mínimo	0,79	0,79		0,79	0,75	
		Máximo	1,10	1,07		1,10	1,07	
Lábio inferior	GC (n=15)	Média	0,91	0,98	0,363	0,90	0,90	0,609
		DP	0,16	0,31		0,16	0,18	
		Mediana	0,89	0,88		0,88	0,86	
		Mínimo	0,63	0,72		0,62	0,72	
		Máximo	1,28	1,88		1,28	1,52	
	GV (n=15)	Média	0,85	0,93	0,691	0,85	0,92	0,955
		DP	0,13	0,25		0,12	0,26	
		Mediana	0,86	0,88		0,84	0,88	
		Mínimo	0,64	0,73		0,65	0,71	
		Máximo	1,09	1,80		1,09	1,80	
	GIV (n=15)	Média	0,94	0,84	0,053	0,94	0,84	0,064
		DP	0,25	0,06		0,26	0,07	
		Mediana	0,91	0,83		0,90	0,82	
		Mínimo	0,53	0,76		0,71	0,76	
		Máximo	1,74	1,03		1,82	1,02	
	GP (n=15)	Média	0,92	0,86	0,570	0,92	0,83	0,281
		DP	0,21	0,09		0,21	0,10	
		Mediana	0,88	0,86		0,87	0,86	
		Mínimo	0,74	0,74		0,74	0,63	
		Máximo	1,59	1,03		1,59	0,99	

* Teste Wilcoxon

**Legenda:** GC = grupo controle; GV = grupo irradiado com *laser* vermelho; GIV = grupo irradiado com *laser* infravermelho; GP = grupo placebo; IFE = índice de fadiga eletromiográfica; TFBM = terapia por fotobiomodulação; DP = desvio padrão; n = número de indivíduos.

A média dos valores de RMS e os valores normalizados pelo pico também não mostraram diferença estatisticamente significativa quando comparados antes e depois da TFBM (Tabela 3).

**Tabela 3:** Comparação da média RMS e os valores de amplitude normalizados pelo pico antes e após fotobiomodulação

Local do eletrodo			Média (RMS)			Valores normalizados pelo pico (%)		
			Antes da TFBM	Após a TFBM	Antes x Após Valor de p^*^	Antes da TFBM	Após a TFBM	Antes x Após Valor de p*
Lábio superior	GC (n=15)	Média	110,10	107,64	0,532	68,94	68,14	0,910
		DP	36,57	52,69		9,53	10,19	
		Mediana	101,94	109,84		69,71	70,64	
		Mínimo	53,92	27,93		51,01	47,96	
		Máximo	176,21	199,43		82,74	84,24	
	GV (n=15)	Média	145,61	138,21	0,733	67,94	65,25	0,470
		DP	87,17	67,60		6,56	7,90	
		Mediana	134,85	116,60		67,25	66,20	
		Mínimo	25,46	37,67		55,44	44,74	
		Máximo	309,66	263,43		77,02	75,54	
	GIV (n=15)	Média	119,80	120,23	0,955	66,05	66,63	0,865
		DP	49,98	47,55		10,11	5,25	
		Mediana	111,86	133,43		67,90	64,96	
		Mínimo	43,21	41,59		45,23	60,07	
		Máximo	195,68	209,90		82,26	75,40	
	GP (n=15)	Média	117,93	115,53	0,496	67,12	64,97	0,394
		DP	79,11	67,10		7,38	9,32	
		Mediana	118,17	122,36		68,19	67,08	
		Mínimo	26,62	21,78		53,07	42,12	
		Máximo	308,36	221,91		77,55	75,51	
Lábio inferior	GC (n=15)	Média	212,51	229,46	0,281	66,58	68,11	0,865
		DP	76,01	66,10		12,02	8,38	
		Mediana	205,19	235,74		70,32	68,42	
		Mínimo	105,24	111,49		31,16	51,11	
		Máximo	364,98	329,42		76,09	80,26	
	GV (n=15)	Média	279,60	269,90	0,701	67,51	66,65	0,638
		DP	131,33	109,33		12,57	8,46	
		Mediana	309,45	238,77		70,98	66,46	
		Mínimo	93,43	117,86		32,64	44,65	
		Máximo	466,10	463,74		78,30	81,33	
	GIV (n=15)	Média	196,12	226,80	0,233	71,40	63,30	0,820
		DP	79,87	91,51		39,17	17,01	
		Mediana	213,34	214,28		64,18	66,97	
		Mínimo	56,58	99,28		18,24	13,30	
		Máximo	321,14	473,99		202,54	79,78	
	GP (n=15)	Média	217,92	212,57	0,650	63,96	65,94	0,865
		DP	132,07	105,65		14,00	7,52	
		Mediana	158,68	218,37		69,50	66,79	
		Mínimo	35,74	64,26		25,26	53,64	
		Máximo	452,33	386,26		78,98	81,62	

* Teste Wilcoxon

**Legenda:** RMS = Root Mean Square; GC = grupo controle; GV = grupo irradiado com *laser* vermelho; GIV = grupo irradiado com *laser* infravermelho; GP = grupo placebo; TFBM = terapia por fotobiomodulação; DP = desvio padrão; n = número de indivíduos.

Também, não foram encontradas diferenças com relevância estatística na análise da comparação entre os lábios superior e inferior antes e após a TFBM (Tabela 4).

**Tabela 4:** Diferença entre a amplitude dos lábios inferior e superior antes e após fotobiomodulação

Local do eletrodo		Diferença entre lábios superior e inferior (RMS)		Valor de p^*^
		Antes da TFBM	Após a TFBM	
GC (n=15)	Média	113,71	124,45	0,394
	DP	69,56	70,33	
	Mediana	99,58	123,57	
	Mínimo	3,91	3,92	
	Máximo	227,76	210,03	
GV (n=15)	Média	148,39	131,68	0,173
	DP	81,71	70,81	
	Mediana	156,60	121,37	
	Mínimo	10,86	16,70	
	Máximo	313,73	254,37	
GIV (n=15)	Média	82,00	111,68	0,307
	DP	56,00	97,40	
	Mediana	94,19	88,46	
	Mínimo	0,45	17,88	
	Máximo	174,05	399,86	
GP (n=15)	Média	127,05	114,93	0,532
	DP	85,15	61,43	
	Mediana	114,49	92,06	
	Mínimo	5,56	24,49	
	Máximo	302,20	202,00	

* Teste Wilcoxon

**Legenda:** RMS = Root Mean Square; GC = grupo controle; GV = grupo irradiado com laser vermelho; GIV = grupo irradiado com laser infravermelho; GP = grupo placebo; TFBM = terapia por fotobiomodulação; DP = desvio padrão; n = número de indivíduos.

## DISCUSSÃO

No presente estudo, não foram observados efeitos imediatos da TFBM na fadiga eletromiográfica do músculo orbicular da boca. Até o momento, não foram encontrados outros estudos que avaliaram os efeitos da fotobiomodulação nessa musculatura.

A variável sexo dos participantes poderia ter influenciado as demais variáveis desse estudo, uma vez que homens usualmente possuem maior força de lábios do que mulheres^(19,20)^. Portanto, optou-se por incluir na amostra somente participantes do sexo feminino. A análise estatística demonstrou que os grupos testados foram homogêneos em relação à idade, fato relevante, pois a força de lábios também sofre influência da idade^(21)^. A dose de 4 J foi escolhida baseada em outros estudos que investigaram os efeitos da TFBM no desempenho de diferentes músculos^(10,22)^.

de Almeida et al.^(23)^ pesquisaram os efeitos do *laser* vermelho (660 nm) e infravermelho (830 nm) no desempenho do músculo bíceps braquial e verificaram que ambos os comprimentos exerceram efeitos positivos. Contudo, somente o *laser* infravermelho influenciou a fadiga. De acordo com os autores, essa diferença resulta do maior alcance de penetração do *laser* infravermelho, que é capaz de atingir fibras musculares mais profundas, enquanto o *laser* com comprimento de onda vermelho atua mais superficialmente^(23)^. A penetração da luz aumenta, de acordo com o aumento do comprimento de onda do *laser* ^(17)^. Por essa razão, o *laser* infravermelho é mais empregado em estudos de análise de desempenho muscular^(13)^. No entanto, como o músculo orbicular da boca é fino^(24)^ e superficialmente localizado^(1)^, considerou-se importante verificar os efeitos do *laser* vermelho, além do infravermelho, neste músculo.

A TFBM, com ambos os comprimentos de onda, não exerceu efeito nos parâmetros relacionados à amplitude do sinal eletromiográfico (RMS e amplitude normalizada), o que concorda com alguns estudos^(25,26)^. da Silva Alves et al.^(25)^ não detectaram efeito do *laser* infravermelho (850 nm) sobre a amplitude do sinal eletromiográfico dos músculos quadríceps e gastrocnêmio de homens jovens e dos Santos Maciel et al.^(26)^ também não encontraram influência da irradiação com *laser* de 780 nm antes dos exercícios de força e resistência no RMS dos músculos tibiais anteriores, medidos durante os exercícios. Muñoz et al.^(27)^, contudo, observaram aumento na amplitude do sinal eletromiográfico após a irradiação com *laser* infravermelho (780 nm) no músculo masseter em homens saudáveis^(27)^, usando uma dose de 0,8 J por ponto em 8 pontos irradiados. As diferenças nos parâmetros dosimétricos e músculos avaliados impossibilitam a comparação dos resultados entre as pesquisas.

A TFBM tem demonstrado resultados positivos na interação com tecidos biológicos, promovendo o aumento na produção de energia celular, devido principalmente à absorção de energia luminosa pelas mitocôndrias, estimulando, assim, a cadeia respiratória^(11)^. Essa interação altera o potencial de redox do citoplasma e acelera o fluxo na cadeia de transporte de elétrons mitocondrial, aumentando a síntese de ATP^(11)^, o que explica o melhor desempenho muscular verificado por alguns estudos após a TFBM^(23,28,29)^. Uma possível explicação para os nossos resultados, inclui o curto período de tempo entre a aplicação do *laser* e a coleta dos dados, sendo insuficiente para a luz interagir com o tecido. Outra explicação pode ser a existência de uma curva dose-resposta relatada nos parâmetros de irradiação. A energia do *laser* aplicada no orbicular da boca pode não ter sido suficiente para melhorar a performance muscular.

No presente estudo a TFBM, independentemente do comprimento de onda, também não exerceu efeito na fadiga eletromiográfica. Esse achado está em consonância com o de alguns estudos^(29,30)^ e contrário a outros^(9,11,16)^. Higashi et al.^(30)^ irradiaram o músculo bíceps braquial de mulheres jovens com *laser* de comprimento de onda de 808 nm antes do protocolo de fadiga e não verificaram efeito do *laser* na fadiga eletromiográfica, bem como Toma et al.^(29)^ mostraram que TFBM com laser de 808 nm, aplicada após exercícios de força para quadríceps em pessoas idosas, não modificou o índice de fadiga eletromiográfica. Contudo, outro estudo^(11)^ verificou que a TFBM (808 nm), realizada imediatamente antes de exercícios para o músculo reto femoral, reduziu significativamente o índice de fadiga eletromiográfica. de Brito Vieira et al.^(9)^ irradiaram o músculo quadríceps femoral entre e após os exercícios com *laser* de 808 nm, e, após dois dias da irradiação, os autores verificaram diminuição na fadiga eletromiográfica para o vasto medial e reto femoral comparado ao grupo placebo, o que sugere que há influência do tempo pós irradiação na diminuição da fadiga muscular.

de Souza et al.^(16)^ verificaram, por meio de dinamometria isocinética, que a TFBM (808 nm) antes do exercício reduziu a fadiga nos flexores plantares do tornozelo de indivíduos saudáveis, mas não alterou a frequência mediana da eletromiografia de superfície. Os autores sugerem que a fonte de ação primária de energia pode estar relacionada à produção de energia dentro das fibras musculares, ao invés da influência do recrutamento neuromuscular. O desenvolvimento da fadiga envolve vários fatores como mudança no recrutamento da unidade motora, diminuição do potencial elétrico de membrana e aumento dos níveis de espécies reativas de oxigênio e de espécies reativas de nitrogênio. A frequência mediana é considerada um bom parâmetro para análise eletromiográfica da fadiga neuromuscular^(16)^. Contudo, ela reflete a taxa de disparo dos potenciais de ação neuromusculares. A falta de diferença na frequência mediana verificada nesse estudo pode estar relacionada à falta de influência da TFBM no padrão de recrutamento neuromuscular.

Avaliou-se ainda, neste estudo, a diferença na amplitude do sinal entre o lábio inferior e superior, pois se pensou que poderia haver um efeito no equilíbrio entre os músculos após a TFBM, mas esse efeito não foi provado. Não foram encontrados estudos que tenham estudado esta variável para comparação.

Constituíram limitações do estudo o curto tempo de descanso entre os exercícios, e entre a irradiação e o exercício e a falta de controle das características anatômicas, como a espessura dos lábios, que poderiam possivelmente influenciar os resultados. Por esse motivo, as comparações foram feitas apenas intragrupo. Sugerem-se mais pesquisas com diferentes doses e que incluam indivíduos com alterações miofuncionais orofaciais, tais como respiradores orais.

## CONCLUSÃO

Não foram encontradas diferenças na eletromiografia de superfície entre as medidas de frequência mediana e amplitude do sinal, realizadas antes e depois da irradiação do orbicular da boca com *laser* de baixa intensidade nos comprimentos de onda 660 nm e 830 nm. Portanto, a fotobiomodulação com os parâmetros avaliados na presente pesquisa não resultou em efeitos imediatos sobre a fadiga no orbicular da boca.
